# A four gene signature predictive of recurrent prostate cancer

**DOI:** 10.18632/oncotarget.13837

**Published:** 2016-12-09

**Authors:** Justin Komisarof, Matthew McCall, Laurel Newman, Wiam Bshara, James L Mohler, Carl Morrison, Hartmut Land

**Affiliations:** ^1^ Departments of Biomedical Genetics, University of Rochester Medical Center, Rochester NY, 14642, USA; ^2^ Biostatistics and Computational Biology, University of Rochester Medical Center, Rochester NY, 14642, USA; ^3^ Department of Pathology, Roswell Park Cancer Institute, Buffalo, NY, 14623, USA; ^4^ Department of Urology, Roswell Park Cancer Institute, Buffalo, NY, 14623, USA; ^5^ Wilmot Cancer Institute, University of Rochester Medical Center, Rochester NY, 14642, USA

**Keywords:** prostate cancer, biochemical recurrence, cooperation response genes, radical prostatectomy, algorithmic prediction

## Abstract

Prostate cancer is the most common form of non-dermatological cancer among US men, with an increasing incidence due to the aging population. Patients diagnosed with clinically localized disease identified as intermediate or high-risk are often treated by radical prostatectomy. Approximately 33% of these patients will suffer recurrence after surgery. Identifying patients likely to experience recurrence after radical prostatectomy would lead to improved clinical outcomes, as these patients could receive adjuvant radiotherapy. Here, we report a new tool for prediction of prostate cancer recurrence based on the expression pattern of a small set of cooperation response genes (CRGs). CRGs are a group of genes downstream of cooperating oncogenic mutations previously identified in a colon cancer model that are critical to the cancer phenotype. We show that systemic dysregulation of CRGs is also found in prostate cancer, including a 4-gene signature (HBEGF, HOXC13, IGFBP2, and SATB1) capable of differentiating recurrent from non-recurrent prostate cancer. To develop a suitable diagnostic tool to predict disease outcomes in individual patients, multiple algorithms and data handling strategies were evaluated on a training set using leave-one-out cross-validation (LOOCV). The best-performing algorithm, when used in combination with a predictive nomogram based on clinical staging, predicted recurrent and non-recurrent disease outcomes in a blinded validation set with 83% accuracy, outperforming previous methods. Disease-free survival times between the cohort of prostate cancers predicted to recur and predicted not to recur differed significantly (p = 1.38×10^-6^). Therefore, this test allows us to accurately identify prostate cancer patients likely to experience future recurrent disease immediately following removal of the primary tumor.

## INTRODUCTION

Prostate cancer is the most common non-dermatological cancer among men in the United States, with an estimated 220,000 new diagnoses in 2015 [1]. Prostate cancer is often indolent and may not require immediate treatment upon diagnosis. On the other hand, prostate cancer can adopt a locally aggressive and rapidly metastatic phenotype that is fatal without intervention. The aggressiveness of prostate cancer can be assessed via clinical staging, levels of prostate-specific antigen (PSA) and the Gleason score, a histological measure of tumor organization. Patients with intermediate or high-risk prostate cancer are often treated by radical prostatectomy, an invasive surgical procedure that removes the prostate in its entirety as well as the pelvic lymph nodes. Radical prostatectomy is potentially curative, but approximately 33% of patients will experience biochemical persistence or recurrence, as defined by a non-zero serum PSA level [2, 3]. These patients may receive salvage radiotherapy, which has demonstrated only modest benefits to survival [4]. Salvage radiotherapy substantially decreases the rate of local recurrence, but many of these patients will develop metastatic disease [5]. Adjuvant radiotherapy has been found to significantly decrease the rate of biochemical recurrence and increase cancer-free survival [6]. However, to prevent over-treatment, adjuvant radiotherapy is typically reserved for patients who have diffusely positive surgical margins or tumor invasion through the prostatic capsule [7]. Predictive methods for identifying patients likely to develop recurrent disease are thus critical for selecting proper treatment.

Most prediction models for prostate cancer recurrence are based on clinical features alone [8]. Gene expression signatures predictive of patient outcomes are in clinical use for many other cancers, and therefore prediction models for prostate cancer may be greatly improved by the inclusion of a molecular component. Previous efforts made to identify genes predictive of prostate cancer recurrence have utilized microarray data, which were comprehensive, but had limited reproducibility [9–15]. We chose to circumvent the microarray reproducibility issue through quantitative PCR analysis of carefully selected smaller gene sets. This also allows us to identify differentially expressed genes by the same method by which gene expression would be determined in the clinic.

Cooperation Response Genes (CRGs) are a group of genes synergistically dysregulated in response to cooperating oncogenic mutations. They are critical to the cancer phenotype at about 50% frequency [16]. Due to their significant influence on the malignant phenotype, we hypothesized that expression of a subset of CRGs might be predictive of prostate cancer recurrence.

In this study, we analyzed CRG expression in a set of frozen prostate cancer samples. A gene signature differentially expressed in prostate cancer that later recurred was identified, and various predictive algorithms were evaluated using this signature. The best algorithm was then combined with a surgical nomogram that further increased predictive power. This was capable of predicting clinical outcomes of an independent blinded validation set with 83% accuracy, outperforming previous methods.

## RESULTS

Tissue from prostate cancer and benign prostate was collected from patients (n = 55) who underwent radical prostatectomy between 1990 and 2002. All patients had newly-diagnosed, clinically localized prostate cancer [17]. Data collected for each patient included standard prognostic variables, such as clinical (cTNM – a measure of tumor size, nodal involvement, and metastasis) and pathological (pTNM) stage, Gleason score, and PSA.

**Figure 1:**
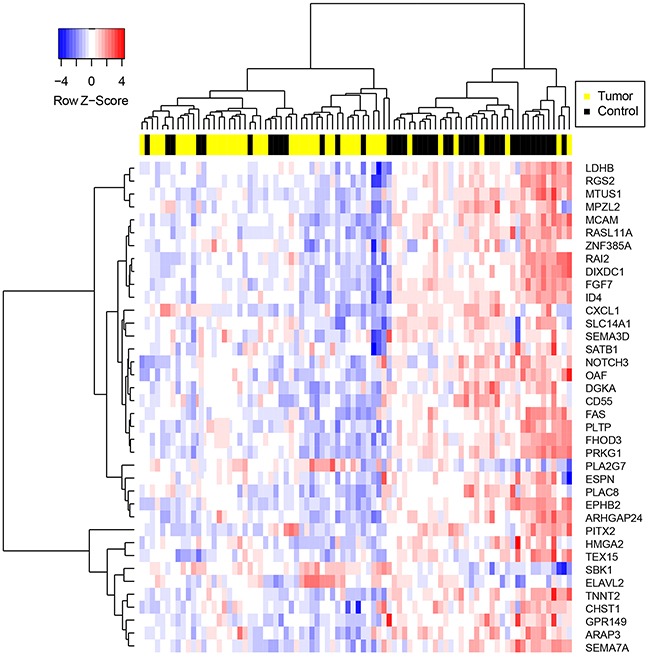
Hierarchical clustering of samples on highly significantly differentially expressed CRGs Genes were selected via two-tailed t-test between prostate cancer and benign tissue specimens with p-value < .01. Gene expression values were determined following normalization of qPCR data and imputation of missing values using the R package “nondetects”.

Differences in CRG expression between prostate cancer and benign prostate were assessed post-normalization. 64% of CRGs were significantly dysregulated in prostate cancer compared to benign prostate (two-tailed t-test, p < .05) (Supplementary Figure S1), and expression values of CRGs distinguished the majority of malignant from benign samples via hierarchical clustering (Figure 1).

The samples were separated into training (n = 32) and validation (n = 23) sets, and disease outcomes were blinded in the validation set. The strategy was to test multiple methods of making predictions using the training set, identify the method that generated the most accurate predictions via cross-validation, and use this method to make predictions using the validation set.

To make predictions on patient outcomes, we first normalized our gene expression data. In addition, a newly developed method for imputing missing gene expression values caused by PCR amplification failure was implemented [18]. Two-tailed t-tests were performed on the normalized training set to identify the genes most differentially expressed between the recurrent and non-recurrent cohorts. Various p-value cutoffs were used to create gene signatures of varying sizes. Finally, three prediction algorithms based on standard clustering techniques were developed and software to implement them was written in *R*.

**Figure 2:**
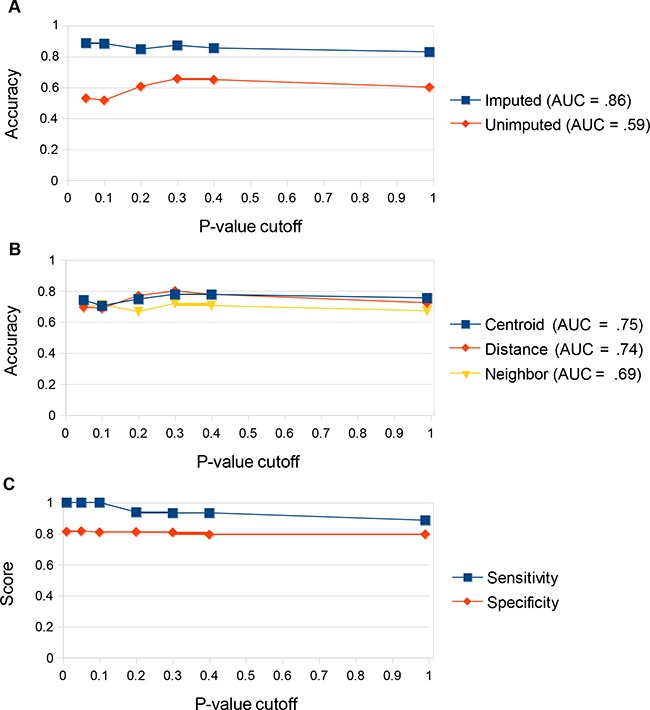
Determining ideal prediction conditions using the training set **a**. Prediction accuracy (% correct predictions) on the training set comparing imputed vs unimputed data. Predictions were made on either imputed or unimputed data via Leave-One-Out Cross Validation (LOOCV) using all three prediction algorithms (centroid, distance, and nearest neighbor) at multiple gene signature p-value cutoffs. The arithmetic mean for accuracy under all three algorithms was displayed for each p-value cutoff. Area under the curve of arithmetic means was computed. Predictions were significantly better across all p-value cutoffs and all algorithms tested using the imputed data. **b**. Prediction accuracy on the training set comparing the three prediction algorithms. Predictions were made using each algorithm via Leave-One-Out Cross Validation (LOOCV) using both imputed and unimputed data at multiple gene signature p-value cutoffs. The arithmetic mean for accuracy under both data-handling techniques was displayed for each p-value cutoff. Area under the curve of arithmetic means was computed. The centroid and distance algorithms were superior to the nearest neighbor algorithm. The centroid algorithm was most effective at reducing the impact of outliers on the data. **c**. Prediction accuracy on the training set comparing p-value cutoffs. Predictions were made using the optimal data handling strategy (normalization to BECN1 and imputation via R package “nondetects”) and prediction algorithm (centroid) at multiple gene signature p-value cutoffs. Prediction accuracy was highest at a p-value cutoff of <0.01, which produced a 4-gene signature. This strategy gave 100% sensitivity for the training set.

Each algorithmic combination was assessed by Leave-one-out cross-validation (LOOCV) using the training data set. Each combination consists of a data handling method, a p-value cutoff for inclusion in gene signature, and a prediction algorithm. Of the data-handling strategies we tested, predictions made using data normalized to BECN1 and then imputed to restore missing values caused by PCR amplification failure performed best, with accuracy of 86% averaged across the three algorithmic predictive methods and p-value cutoffs (Figure 2a). Predictions made with unimputed data had an accuracy of only 59% (Figure 2a). Of the three prediction algorithms tested, the *centroid* algorithm resulted in the most accurate predictions, with an accuracy of 75% averaged across all data handling conditions and p-value cutoffs (Figure 2b). The *distance* algorithm also performed well (accuracy = 74%) on the training set, while the *nearest neighbor* algorithm was inferior (accuracy = 69%) (Figure 2b). The highest overall accuracy of 90% was achieved using the lowest p-value cutoff (p < 0.01) (Figure 2c). This cutoff resulted in the generation of a 4-gene signature (Table 1). We therefore chose to make predictions on the validation set using the *centroid* algorithm with a 4-gene signature derived from applying a p-value cutoff of <0.01 to the BECN1-normalized and imputed data. Predictions made on the training set using these conditions had a sensitivity of 100% (Figure 2c). The lack of false negatives is important since they represent patients who could benefit from adjuvant radiotherapy but would not receive it based on the predictions.

**Table 1:** CRG predictive gene signature with fold changes in recurrent samples

Gene	Fold change in recurrent
HBEGF	-2.2
HOXC13	6.7
IGFBP2	-1.4
SATB1	-3.1

Genes chosen were differentially expressed between recurrent and non-recurrent cohorts using two-tailed t-test for independent samples with unequal variance with p-value < 0.01.

**Figure 3:**
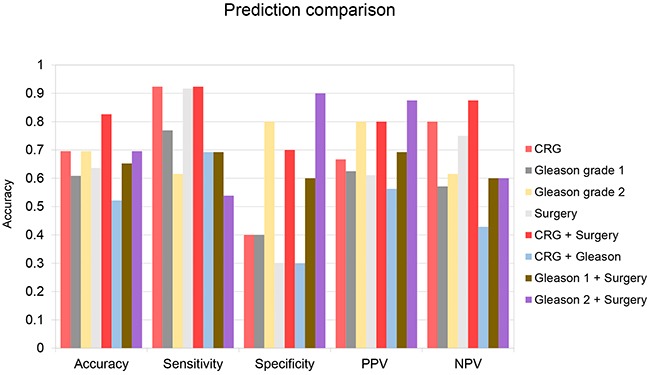
Test set prediction data Predictions were made using 5 different prediction strategies. Strategy 1: Use centroid prediction algorithm and 4-gene CRG signature comparing samples to training set data normalized to BECN1 and imputed. (CRG) Strategy 2: Samples were predicted to be recurrent if Gleason score was 7-10 and non-recurrent otherwise. (Gleason 1) Strategy 3: Samples were predicted to be recurrent if Gleason score was 7 (4+3), or above, and non-recurrent if Gleason score was 7 (3+4) or below. (Gleason 2) Strategy 4: Samples were predicted to be non-recurrent if primary tumor staging was <T2c, lymph node stage was 0 and metastatic staging was 0 and recurrent otherwise. (Surgery) Strategy 5: Samples were predicted to be non-recurrent if primary tumor staging was <T2c, lymph node stage was 0 and metastatic staging was 0. Other samples were evaluated using strategy 1. (CRG + Surgery) Strategy 6: Samples were predicted to be recurrent if Gleason score was 9-10 and non-recurrent if Gleason grade was 2-6. Other samples were evaluated using strategy 1. (CRG + Gleason) Strategy 7: Samples were predicted to be non-recurrent if primary tumor staging was <T2c, lymph node stage was 0 and metastatic staging was 0. Other samples were evaluated using strategy 2. (Gleason 1 + Surgery) Strategy 8: Samples were predicted to be non-recurrent if primary tumor staging was <T2c, lymph node stage was 0 and metastatic staging was 0. Other samples were evaluated using strategy 3. (Gleason 2 + Surgery) Accuracy = % predictions correct. Sensitivity = (1 - %False Negatives). Specificity = (1 - %False Positives). PPV = (# Recurrent tumors/# Total Recurrent Predictions). NPV = (# Non-recurrent tumors/# Total Non-Recurrent Predictions).

Our chosen conditions accurately predicted 70% of the samples in our validation set, outperforming or equaling two sets of predictions made utilizing different cutoffs of Gleason score (61%, 70%) and predictions made based on surgical stage (64%) (Figure 3). For our CRG-based predictions, sensitivity (92%) was significantly higher than specificity (40%), indicating that we were successfully identifying recurrent tumors, but also misidentifying many non-recurrent tumors as likely to recur. We hypothesized that these errors may result from making predictions about prostate cancers with an unfavorable gene expression profile, but which nevertheless were small, located in only one half of the prostate, with no lymph node involvement, no distant metastases, and negative margins on surgery. These cancers were detected early and cured by surgery. Therefore, surgical information was incorporated into the CRG prediction decision procedure to improve specificity. Using this modified decision procedure, prediction accuracy improved to 83% (Figure 3). Specificity improved to 70%, while our sensitivity remained high at 92% and false negatives were few. In comparison, predictions based on the CRG signature were not improved when combined with predictions based on Gleason grade (Figure 3). Likewise, predictions made using Gleason grade were not improved when combined with surgical information (Figure 3). In our most important metrics, accuracy and sensitivity, the CRG + surgery-based predictions outperformed all other prediction modalities.

**Figure 4:**
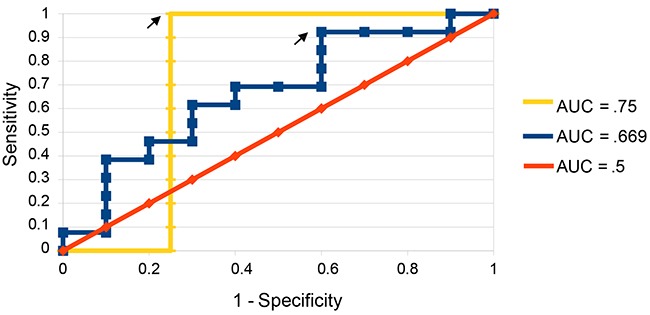
ROC curves for predictions on validation set ROC curves were constructed both with all samples considered (blue) and with those best handled by surgical predictions removed compared to predictions made by chance (yellow). Arrows indicate the points at which recurrence score and non-recurrence score were given equal weight and predictions were made.

Receiver operating characteristic (ROC) curves were created to evaluate the sensitivity and specificity of predictions made using different discrimination thresholds for recurrence or non-recurrence. ROC curves generated using CRG-based predictions resulted in an area under the curve (AUC) of 0.67 (Figure 4). When samples best handled by predictions based on surgical information were removed from the sample set, the AUC increased to 0.75 (Figure 4).

**Figure 5:**
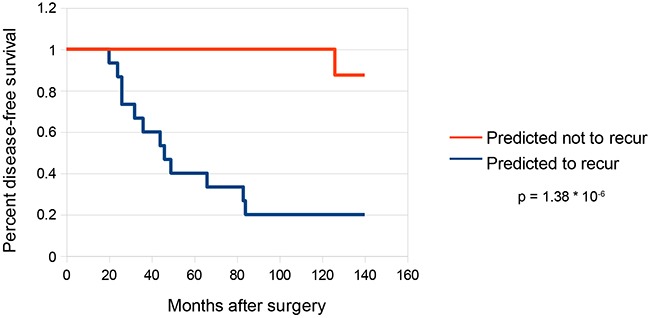
Survival curves for predictions on validation set Kaplan-Meier survival curves were constructed for the subset of samples in the validation set that were predicted to recur (n = 15) as well as the subset of samples predicted not to recur (n = 8). Statistical analysis was performed by log-rank test using the R package “survdiff”.

Kaplan-Meier survival curves were created to visualize recurrences in our predicted high risk and low risk cohorts (Figure 5). Of the patients in the validation set predicted to recur (n = 15), 12 experienced biochemical recurrence at a median time of 40 months post-prostatectomy. Only one patient in the cohort predicted not to recur (n = 8) experienced biochemical recurrence 126 weeks post-prostatectomy, a highly significant result (p= 1.38×10^-6^, log-rank test).

To control for the possibility that the predictive power of the gene signature reported here may be affected by variation in stromal content of the prostate cancer specimens, we assessed the mRNA expression of two stromal markers, smooth muscle alpha-actin and vimentin, in our validation set. The two mRNAs are highly expressed in smooth muscle, fibroblasts, and myofibroblasts. Neither vimentin nor alpha-actin was significantly differentially expressed in recurrent vs non-recurrent cohorts using both non-normalized expression values (p = .18, .95, two-tailed student's t-test) (Supplementary Figure S2a) and expression values normalized to Becn1 mRNA (p = .88, .15) (Supplementary Figure S2b). Consistently, Becn1 mRNA was also not significantly differentially expressed in the two cohorts (p = .69) (Supplementary Figure S2a). In addition, expression levels for Becn1 and RhoA mRNAs, and ribosomal 18S RNA were strongly correlated between all samples (r = .822, .658, .850, Pearson's correlation coefficient) (Supplementary Figure S3), suggesting that Becn1 expression qualifies as a reasonable reference for sample normalization. Taken together, these results suggest that the predictive power of the gene signature originates primarily from gene expression differences associated with cancer cells, rather than variance in the stromal content of tissue samples.

## DISCUSSION

An estimated 50,000 radical prostatectomies are performed each year in the US, and about 15,000 of these patients will experience biochemical recurrence. Here we show that a four-gene signature based on HBEGF, HOXC13, IGFBP2, and SATB1 was able to identify patients whose prostate cancer recurred. The prediction algorithm that incorporated surgical information provided accurate prognostic information on patient outcomes with 83% accuracy. Most patients undergoing radical prostatectomy receive no additional treatment beyond regular monitoring of PSA. Adjuvant radiotherapy administered immediately after radical prostatectomy could improve outcomes for patients identified using the CRG signature to be at high risk for recurrence.

The four genes identified in our signature, HBEGF, HOXC13, IGFBP2, and SATB1, all play significant roles in the modification of cancer phenotypes. HOXC13, a homeobox-family transcription factor known to control cell proliferation and differentiation, was found to be upregulated in our recurrent prostate cancer samples. HOXC13 has been reported to be upregulated in metastatic melanoma compared to primary tumor tissue [19]. Furthermore, knockdown of HOXC13 has been reported to decrease viability of several cancer cell lines *in vitro*, including the prostate cancer line PC-3ML [20]. SATB1 is a chromatin organizer responsible for the recruitment of chromatin remodeling proteins, and was found to be downregulated in our recurrent sample cohort. In colorectal cancer, loss of SATB1 was reported to be a strong predictor of worse outcomes [21]. Publicly available microarray data indicates that SATB1 is downregulated in prostate cancer as well as other solid tumors. Increased nuclear localization of SATB1 has been reported to be correlated with increased prostate cancer aggression and invasive potential [22]. Although this runs counter to our observation, it is possible that our recurrent prostate cancer samples may have higher nuclear localization despite lower overall expression. The appearance of IGFBP2 in our gene signature was reassuring, since decreased expression of this gene has been reported to be a predictor of prostate cancer recurrence after radical prostatectomy [23]. The structurally similar protein IGFBP5 has also been found to be downregulated in metastatic prostate cancer [24]. Finally, HBEGF is a ligand of the EGF receptor that also binds heparin. HBEGF has not been studied in prostate cancer, but HBEGF is known to promote invasion and metastasis in breast, colon, and ovarian cancer [25, 26, 27]. HBEGF was unexpectedly downregulated in the recurrent prostate cancer samples, which would appear at odds with its previously documented role in cancer. However, a recent study found that the expression of the growth factor receptor FGFR1 was associated with indolent prostate cancer. While the particular mechanism by which FGFR1 acts to drive this outcome is unknown, HBEGF may function in a similar way [28].

The focus for this study was biochemical recurrence after radical prostatectomy. Biochemical recurrence after prostatectomy almost always requires the primary tumor to already have escaped the prostatic capsule, either invading local tissue or metastasizing to regional lymph nodes or distant organs. These behaviors are hallmarks of tumor aggressiveness, and suggest that the CRG signature may provide valuable information for assessing patient outcomes even in patients who have not undergone radical prostatectomy. Currently the best test to determine risk of aggressive disease is the Gleason score, a test with limited predictive power in many cases. Several groups have already identified gene signatures that are hallmarks of either indolent or aggressive disease [24, 28, 29]. The CRG signature, which is predictive of biochemical recurrence of prostate cancer, could likewise provide useful prognostic information to patients at the point of diagnosis, and further tests should be conducted to evaluate this possibility. This would, however, necessitate the use of formalin-fixed, paraffin-embedded biopsy tissue.

Irshad et al. [28] identified a three-gene signature associated with aging and cellular senescence that is predictive of indolence in prostate cancer with low Gleason grades. These low-grade cancers are often managed with active surveillance, however, not all of them are indolent. Identifying patients who would typically not be treated aggressively who do in fact require an intervention is also the focus of our study. Likewise, Ross-Adams et al. [30] identified a 100-gene panel predictive of prostate cancer recurrence using a transcriptomics approach. The indolence signature and the transcriptomics signature may complement the recurrence signature reported herein; combining these different approaches may lead to better prediction of prostate cancer outcomes.

The success of this model to predict prostate cancer recurrence speaks to the importance of the CRGs in regulating human cancer behavior. As the CRGs were originally identified in a colon cancer background, it stands to reason that CRG expression may provide valuable prognostic information for colon cancer as well. Early-stage colon cancer is treated primarily with surgical resection, and assessing the likelihood of recurrence is an important clinical question. Future studies of the predictive potential of CRGs may provide greater insight into the likelihood of different disease outcomes, thus permitting better-informed decisions about treatment.

## MATERIALS AND METHODS

### Tissue specimens

All tissue samples were collected from radical prostatectomy specimens by the RPCI Pathology Resource Network with IRB approval. Patient demographic, clinical, pathology, and outcome data were collected through the Clinical Data Network, another shared resource at RPCI. All tissue specimens were immediately processed and snap frozen in liquid nitrogen within 30 minutes of prostatectomy by the Department of Urology.^17^ All tissue samples were reviewed by a board certified anatomic pathologist to verify the diagnosis of prostatic adenocarcinoma and to estimate the percent neoplastic tumor nuclei. Data collected for each patient included standard prognostic variables, such as Gleason score, clinical (cTNM) and pathological (pTNM) stage, and PSA. All patients had at least 3 years of follow-up data. Biochemical progression was defined by the AUA guidelines of serum PSA of 0.2 ng/mL or greater (obtained 6 weeks - 3 months postoperatively), with a second confirmatory level of PSA greater than 0.2 ng/mL.

### CRG expression data

Total RNA was harvested from frozen tissue sections using a standard Trizol (Life Technologies, Carlsbad, CA) application. Tissues were homogenized with Trizol reagent. RNA was precipitated from the aqueous phase using isopropanol and rehydrated using DEPC water. An RNA aliquot was run on an Agilent 2100 bioanalyzer to confirm RNA integrity by generating a RNA Integrity Number (RIN) value. RNA was converted to cDNA and quantified via Taq-Man Low Density Array (TLDA) RT-PCR.

### Data handling

Ct values were normalized to Becn1. Non-detects were imputed based on estimation of a non-random missing data mechanism using the nondetects R/Bioconductor package.^18^ Statistical assessment of differential expression was performed using a t-test based on maximum likelihood estimates (MLEs) of the within group means and variances generated by the development version of the nondetects package (manuscript in preparation).

### Generation of predictions

Two-tailed t-tests were performed on normalized data to identify genes differentially regulated between biochemically recurrent and non-recurrent cohorts. Multiple p-value cutoffs were tested to assess the relative success of different sizes of gene signature. Three prediction algorithms based on clustering techniques were generated and software was written to implement each in R. Algorithms incorporated a gene signature of size N and evaluated samples as points in N-dimensional space. The “distance” algorithm generates recurrence and non-recurrence scores by comparing the Euclidian distance between the sample point and all points in the recurrent and non-recurrent groups respectively. The “centroid” algorithm generates recurrence and non-recurrence scores by comparing the distance between the sample point and the centroids of the recurrent and non-recurrent group. The “nearest neighbor” algorithm generates recurrence and non-recurrence scores by comparing the distance between the sample point and the closest member in both the recurrent and non-recurrent groups. Predictions are made by selecting the lower of either the recurrence or non-recurrence scores.

### Algorithms

#### Distance

$$d=\overset{n}{\underset{1}{\sum }}\sqrt{{\left(nr_{\left(n_{1}\right)}-x_{\left(1\right)}\right)}^{2}+{\left(nr_{\left(n_{2}\right)}-x_{\left(2\right)}\right)}^{2}+\ldots +{\left(nr_{\left(n_{i}\right)}-x_{\left(i\right)}\right)}^{2}}$$

recurrence score

$$d=\overset{n}{\underset{1}{\sum }}\sqrt{{\left(r_{\left(n_{1}\right)}-x_{\left(1\right)}\right)}^{2}+{\left(r_{\left(n_{2}\right)}-x_{\left(2\right)}\right)}^{2}+\ldots +{\left(r_{\left(n_{i}\right)}-x_{\left(i\right)}\right)}^{2}}$$

non-recurrence score

I = # genes in signature

N = # samples in training set

R_n(i)_ = expression of the i^th^ gene in the signature in the n^th^ recurrent sample in the training set

NR_n(i)_ = expression of the i^th^ gene in the signature in the n^th^ non-recurrent sample in the training set

X_i_ = expression of the i^th^ gene in the signature in the current sample in the test set

#### Centroid

$$d=\sqrt{{\left({\left(\bar{r}\right)}_{\left(n_{1}\right)}-x_{\left(1\right)}\right)}^{2}+{\left({\left(\bar{r}\right)}_{\left(n_{2}\right)}-x_{\left(2\right)}\right)}^{2}+\ldots +{\left({\left(\bar{r}\right)}_{\left(n_{i}\right)}-x_{\left(i\right)}\right)}^{2}}$$

recurrence score

$$d=\sqrt{{\left({\left(n\bar{r}\right)}_{\left(n_{1}\right)}-x_{\left(1\right)}\right)}^{2}+{\left({\left(n\bar{r}\right)}_{\left(n_{2}\right)}-x_{\left(2\right)}\right)}^{2}+\ldots +{\left({\left(n\bar{r}\right)}_{\left(n_{i}\right)}-x_{\left(i\right)}\right)}^{2}}$$

non-recurrence score

I = # genes in signature

N = # samples in training set

R_n(i)_ = average expression of the i^th^ gene in the signature in the n^th^ recurrent sample in the training set

NR_n(i)_ = average expression of the i^th^ gene in the signature in the n^th^ non-recurrent sample in the training set

X_i_ = expression of the i^th^ gene in the signature in the current sample in the test set

#### Nearest-neighbor

recurrence score

$$d=\sqrt{{\left(r_{\left(1\right)}-x_{\left(1\right)}\right)}^{2}+{\left(r_{\left(2\right)}-x_{\left(2\right)}\right)}^{2}+\ldots +{\left(r_{\left(i\right)}-x_{\left(i\right)}\right)}^{2}}$$

non-recurrence score

$$d=\sqrt{{\left(nr_{\left(1\right)}-x_{\left(1\right)}\right)}^{2}+{\left(nr_{\left(2\right)}-x_{\left(2\right)}\right)}^{2}+\ldots +{\left(nr_{\left(i\right)}-x_{\left(i\right)}\right)}^{2}}$$

I = # genes in signature

R_n(i)_ = expression of the i^th^ gene in the signature in the closest recurrent sample in the training set

NR_n(i)_ = expression of the i^th^ gene in the signature in the closest non-recurrent sample in the training set

X_i_ = expression of the i^th^ gene in the signature in the current sample in the test set

### Evaluation of algorithms on training set

Predictions were made on a 32-sample training set with 16 biochemically recurrent and 16 non-recurrent tumors. Each permutation of normalization method, p-value cutoff for gene signature, and prediction algorithm was evaluated using Leave-one-out cross-validation (LOOCV).

### Evaluation of algorithms on validation set

The centroid algorithm was used to make predictions about a 23-sample validation set using the imputed and BECN1-normalized data with a CRG signature generated using a p-value cutoff of < 0.01.

### Incorporation of surgical information and final predictions

Surgical information was incorporated into the prediction decision procedure to improve specificity. Tumors with clinical stage of T2bN0M0 or below, with negative surgical margins were classified as non-recurrent. All other predictions were generated algorithmically as before.

### ROC curve generation

ROC curves were created using predictions made by varying the discrimination threshold between a prediction of recurrence vs non-recurrence. The model is altered by adding a modifier to the recurrence score before making predictions. When a modifier of -10 is added, all samples were predicted to be non-recurrent; when a modifier of +10 is added, all samples were predicted to be recurrent. Sensitivity and specificity of predictions were measured and plotted for each value of the modifier at which a prediction changes.

### Kaplan-Meier survival curve generation

Kaplan-Meier survival curves were taken by plotting time from prostatectomy to biochemical recurrence for patients predicted to recur and patients predicted not to recur respectively. Statistical analysis was done via log-rank test using the R package “survdiff”.

## SUPPLEMENTARY MATERIALS FIGURES AND TABLE




