# Supplementary material for: A four gene signature predictive of recurrent prostate cancer
Source: Oncotarget. 2016 Dec 9;8(2):3430–40. doi: 10.18632/oncotarget.13837 (PMC5356893; doi:10.18632/oncotarget.13837)
Supplement: Supplementary file 1 [file oncotarget-08-3430-s001.pdf]

A four gene signature predictive of recurrent prostate cancer

SUPPLEMENTARY FIGURES TABLE

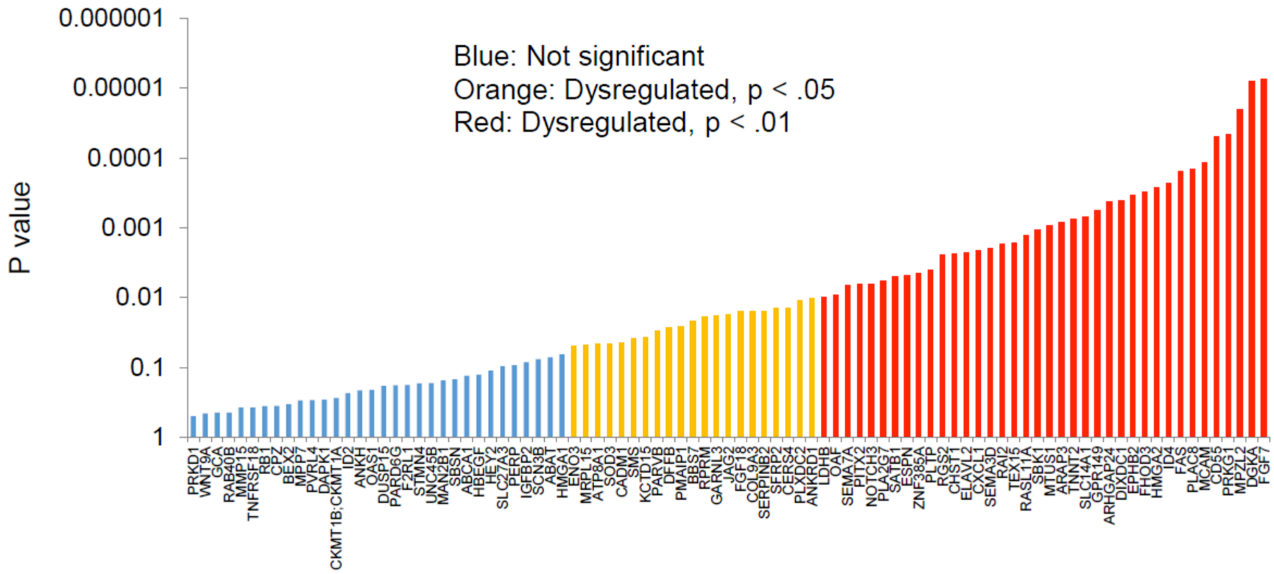

Supplementary Figure S1: Waterfall plot of CRG dysregulation in prostate cancer vs matched benign tissue. Two-tailed t-tests were performed on each CRG and t-statistics were plotted. 58 of 91 CRGs (64%) were significantly dysregulated in prostate cancer ($p < .05$). Of these, 37 CRGs were highly significantly dysregulated ($p < .01$).

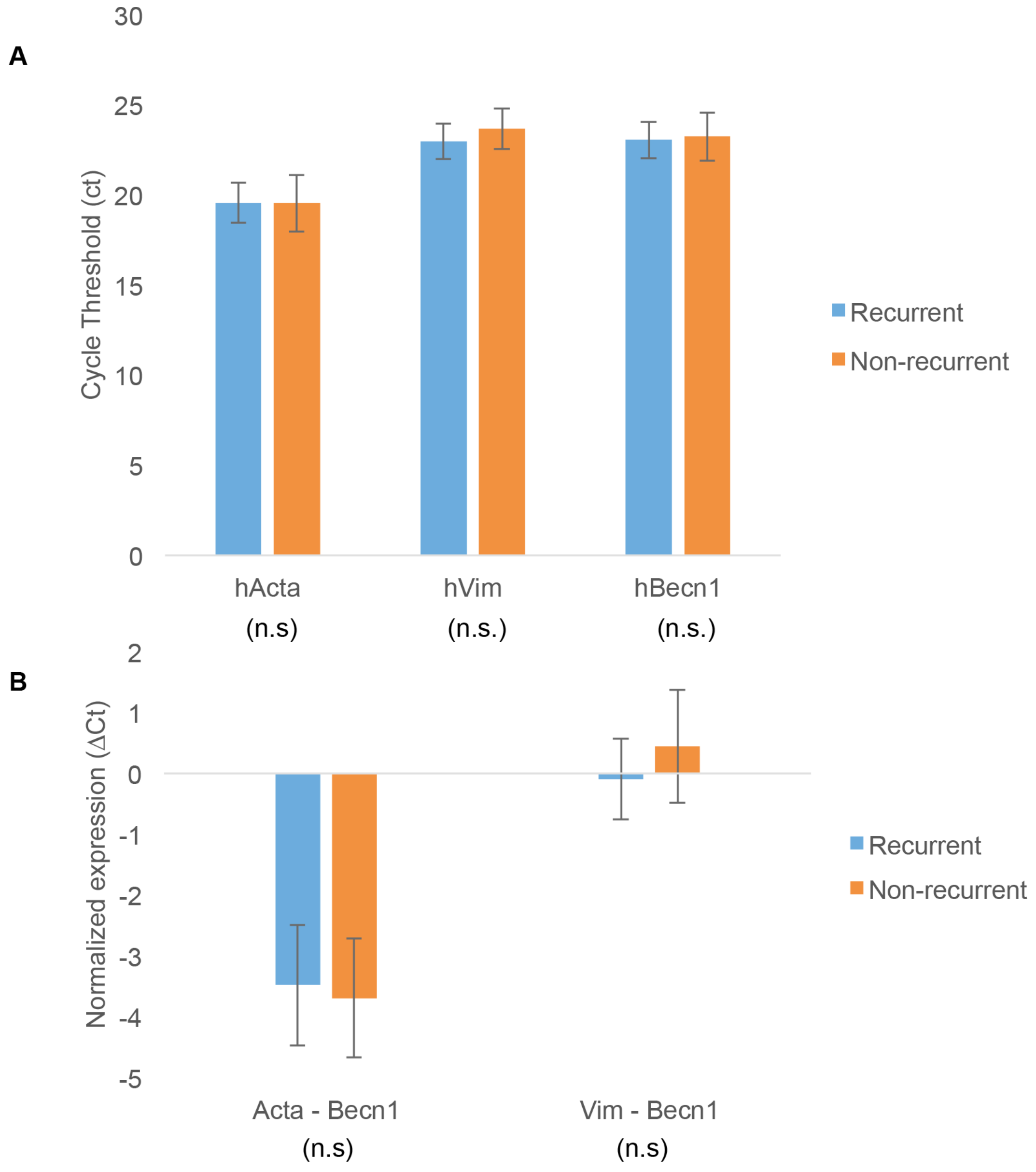

Supplementary Figure S2: Expression of stromal markers in validation set samples. A. Non-normalized expression of smooth muscle alpha-actin (ACTA1), vimentin (VIM), and beclin-1 (BECN1). Expression was quantified via RT-PCR. **B.** Relative expression of smooth muscle alpha-actin and vimentin normalized to beclin-1.

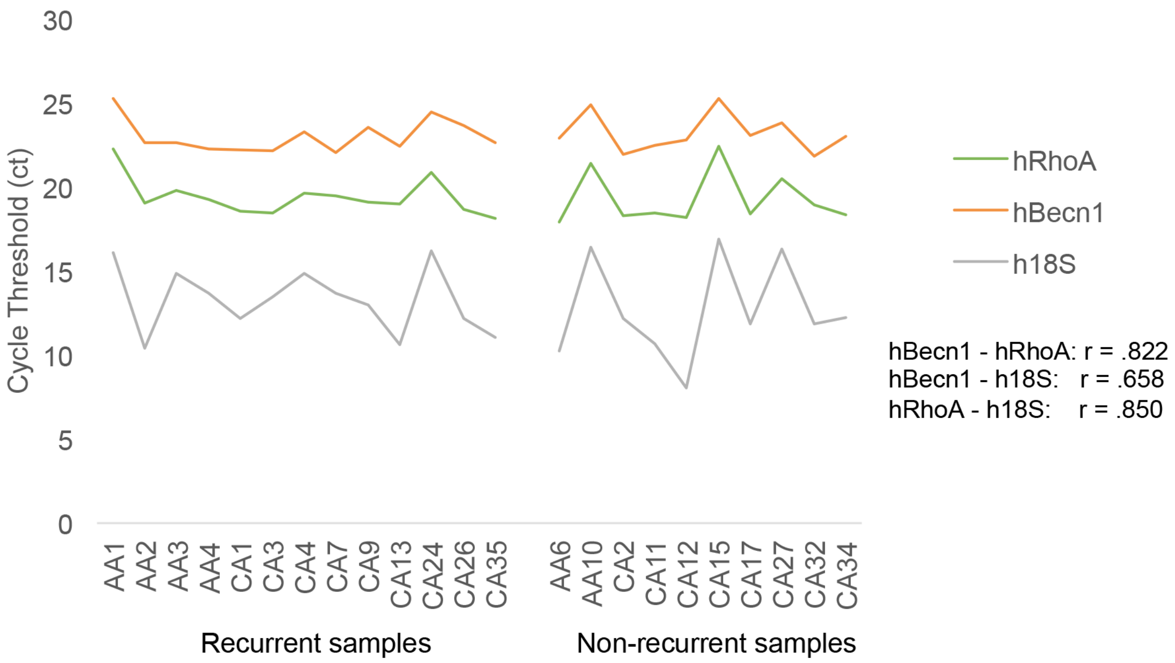

Supplementary Figure S3: Expression of reference genes in validation set samples. Expression of reference genes beclin-1 (BECN1), Ras homolog gene family member A (RHOA), and 18S ribosomal RNA (18S) was quantified via RT-PCR. Pearson's correlational coefficients were calculated between these three genes using expression across all samples in the validation set.

Supplementary Table S1: RT-PCR data derived from human prostate cancer specimens. Raw data represent threshold cycle (Ct) values generated by Taq-Man Low Density Array (TLDA) RT-PCR. Data in sheets 3 and 4 have been normalized to BECN1. In the sheet "Training set normalized data", the data has also been imputed using the "nondetects" R package as described in Methods.

See Supplementary File 1
